# Supplementary material for: Origin of giant electric-field-induced strain in faulted alkali niobate films
Source: Nat Commun. 2022 Jul 7;13:3922. doi: 10.1038/s41467-022-31630-8 (PMC9262982; doi:10.1038/s41467-022-31630-8)
Supplement: Supplementary file 1 — Supplementary Information [file 41467_2022_31630_MOESM1_ESM.pdf]

Supporting Information

# Origin of Giant Electric-Field-Induced Strain in Faulted Alkali Niobate Films

**Moaz Waqar[1,2,3†], Haijun Wu[4†], Khuong Phuong Ong[5], Huajun Liu[2], Changjian Li[1], Ping Yang[1,6], Wenjie Zang[1], Weng Heng Liew[2], Caozheng Diao[6], Shibo Xi[7], David J. Singh[8], Qian He[1], Kui Yao[2,3]*, Stephen J. Pennycook[1,3]*, John Wang[1,2,3]***

[1]Department of Materials Science and Engineering, National University of Singapore, Singapore 117574

[2]Institute of Materials Research and Engineering (IMRE), A*STAR (Agency for Science, Technology and Research), Singapore 138634

[3]Integrative Sciences and Engineering Programme, National University of Singapore, Singapore 119077

[4]State Key Laboratory for Mechanical Behavior of Materials, Xi'an Jiaotong University, Xi'an, China

[5]Institute of High Performance Computing, A*STAR (Agency for Science, Technology and Research), Singapore 138632

[6]Singapore Synchrotron Light Source (SSLS), National University of Singapore, Singapore 117603

[7]Institute of Chemical and Engineering Sciences, A*STAR (Agency for Science Technology and Research, Singapore 627833

[8]Department of Physics and Astronomy and Department of Chemistry, University of Missouri, USA, 65211

[†]These authors contributed equally to this work.
*Corresponding Authors

For correspondence:
k-yao@imre.a-star.edu.sg; stephen.pennycook@cantab.net; msewangj@nus.edu.sg

Keywords: potassium sodium niobate, planar faults, thin film, electromechanical response, piezoelectricity

**Supplementary Section I: Comparison with NPR-NNO film**

The higher electromechanical response of PF-KNN film compared to $NaNbO_3$ film with nano-pillar regions (NPR-NNO)[1] can be attributed to the introduction of K and the higher density of PFs. Firstly, according to our experimental results and analyses, a higher density of PFs results in a larger electromechanical response. It can be visually observed from Fig. 1c in the main text that the network of PFs is denser in PF-KNN as compared to NPR-NNO[1] which contributes to a higher electromechanical response in PF-KNN (1909 pm $V^{-1}$ compared to that of 1098 pm $V^{-1}$ in the case of NPR-NNO). Secondly, the density of PFs in Fig. 1a (0.13 $nm^{-1}$) is close to that reported earlier in NPR-NNO, however, it shows a higher maximum $d^*_{33,f}$ of 1347 pm $V^{-1}$ (at 90 kV $cm^{-1}$) at 1 kHz compared to that of 1098 pm $V^{-1}$ (at 125 kV $cm^{-1}$). The former also shows an electric-field-induced strain of ~1.2 % at 90 kV $cm^{-1}$ compared to ~0.5 % achieved in the latter at the same electric field. Hence, the chemical composition and the induced PFs play a significant role in the observed electromechanical response. In the literature, it is established that $(K,Na)NbO_3$ solid solutions are ferroelectric in nature and show superior piezoelectric properties compared to either of the parent components i.e., $NaNbO_3$ or $KNbO_3$.[2] Bulk $NaNbO_3$ has an antiferroelectric phase, without piezoelectric property. Due to its bigger size, the substitution of Na by K atoms in the niobate system disfavors antiferroelectric $NbO_6$ tilts and rotations while stabilizing the ferroelectric phase[3,4]. Moreover, the geometrical frustration driven by the Na/K size mismatch causes local fluctuations in the Nb-O bond lengths and tilt patterns which result in the softening of lattice and enhanced polarization rotation under external electric field[5,6]. This largely improves the ferroelectric and piezoelectric response[7] and could explain the higher electromechanical response in PF-KNN film compared to NPR-NNO film, even in the case with similar defect density.

**Supplementary Section II: O-K edge as an indicator of Nb valence**

The valence of Nb ions was qualitatively obtained from the O K-edge using the following method: In the perovskite structure of KNN, oxygen atoms in the octahedron are covalently bonded with the Nb atom where O 2*p* orbitals are hybridized with 4*d* orbitals of Nb[8]. Each Nb atom is surrounded by an oxygen octahedron in the perovskite phase which causes the splitting of degenerate 4*d* orbitals into $e_g$ and $t_{2g}$ orbitals with the former having slightly higher energy[9]. The oxygen K loss edge is characterized by 1*s* electron transition to 2*p* unoccupied shell where the peaks *a* and *b* (Fig 2h in the main text) correspond to the $t_{2g}$ and $e_g$ components of the Nb 4*d* – O 2*p* orbital overlapping respectively[8]. The transition from $d_0$ configuration in $Nb^{5+}$ to $d_1$ configuration in $Nb^{4+}$ (or even lower valence) involves the filling of $t_{2g}$ orbitals, which are lower in energy, hence decreasing the $t_{2g}/e_g$ ratio. This decrease is also reflected in the *a*/*b* ratio in the O K-edge hence it is a reliable indicator of valence change of Nb in our case[8,10] and shows a partial filling of 4*d* orbital. The results are further supported by XAS measurements shown in Supplementary Fig. 8 where the same features can be found in the O K-edge spectrum of PF-KNN film accompanied by the Nb-$M_{2,3}$ edge spectrum further validating the lowering of Nb valence.

**Supplementary Section III: Calculation of activation energies**

The activation energy $E_a$ was calculated using the Nernst-Einstein equation[11]

$$\sigma T = \sigma_0 \, e^{\frac{-E_a}{kT}} \qquad \text{Eq. S1.}$$

where $\sigma_0$ is the exponential factor, $k$ is the Boltzmann constant and $\sigma$ is the conductivity at temperature $T$. Hence the slope of ln($\sigma T$)-1000/$T$ plot can be used to determine the $E_a$ for defect migration. $E_a$ can also be calculated using relaxation time, $\tau$, instead of $\sigma$ using the Arrhenius equation. Since polaronic charges show temperature-dependent relaxation at low frequencies ($10^0$ – $10^3$ Hz)[12] similar to our case (Supplementary Fig. 10b), we calculated the activation

energy, $E_a$., using relaxation time $\tau$ as a function of temperature. The obtained value of 0.33 eV is close to the $E_a$ values of bound polarons derived from the first-principles calculations for $ABO_3$ perovskites[13–15] and experimentally observed in potassium sodium niobate ceramics[16]. This suggests that the electrons at the PFs are indeed localized as polarons instead of being free charge carriers.

The dc conductivity ($\sigma_{dc}$) in Supplementary Fig. 11 was calculated by fitting the $\sigma_{ac}$ using Jonscher's power law[17]:

$$\sigma_{ac}(\omega) = \sigma_{dc} + A\omega^n \qquad \text{Eq. S2.}$$

where $\omega$ is the angular frequency and $A$, $n$ are material-dependent constants. This power law is termed Universal Dielectric Response and can be used to model the charge transport of localized charge carriers including the hopping mechanism. The low-frequency AC conductivity response of stochiometric KNN film in the measured temperature range can only be loosely fitted using power law (Supplementary Fig. 11a). However, on the contrary, the response of PF-KNN can be well fitted by the power law (Supplementary Fig. 11c) indicating hopping dominated conduction mechanism which is characteristic of localized charge carriers.

Since $\sigma$ is proportional to the concentration of mobile charge carriers $n$ and their mobility $\mu_e$ i.e., $\sigma = ne\mu_e$ where $e$ is the unit charge, Eq. S1 can be modified as

$$nT = n_0 \, e^{\frac{-E_a}{kT}} \qquad \text{Eq. S3.}$$

where $n_0$ is the carrier concentration when $\frac{10^3}{T} \rightarrow 0$.

According to Eq. S3, $n$ i.e., the concentration of mobile polarons, varies inversely with $E_a$.

**Supplementary Section IV: Polarization vector mapping and domain structure**

The in-plane polarization vector, $\delta_{Nb\_xy}$, (Fig. 2e in the main text) has been calculated by measuring the displacement of Nb atoms with respect to the corner A-site atoms (Na/K). In such a case, the orientation of local spontaneous polarization, $P_s$, should be the same as $\delta_{Nb\_xy}$ whereas the magnitude can be estimated by using $P_s = \kappa\,\delta_{Nb\_xy}$, where $\kappa$ is a material-dependent constant[18]. At the PF interface, the $\delta_{Nb\_xy}$ has been measured relative to the two Na/K atoms from the original cell and the two Nb atoms from the unit cell across the PF interface. This gives a reliable $P_s$ orientation which is consistent with the results obtained when $\delta_{Nb\_xy}$ is measured using oxygen octahedron as standard i.e., $\delta_{NbO\_xy}$ making tail-tail configuration at the interface (Supplementary Fig. 12). However, $P_s$ magnitude at the PF interface might be different from $\kappa\,\delta_{Nb\_xy}$.

The polarization vector mapping from a comparatively larger area is shown in Supplementary Fig. 14. The polarization vectors represented by the shades of red show polarization with a large in-plane component and the ones represented by the shades of blue represent polarization with a small in-plane component. It is evident from the STEM result shown in Supplementary Fig. 14 that the matrix perovskite KNN region (away from the PFs) shows an out-of-plane polarization and a small in-plane component of the polarization vector which conforms with a tetragonal symmetry as is also supported by the XRD results in Supplementary Fig. 4. However, the lattice in the vicinity of PFs has a larger in-plane component with a local symmetry distortion leading to lower symmetry rhombohedral and orthorhombic phases with the possible existence of monoclinic bridging phases (Methods). Further analysis of polarization magnitude and angle given in Supplementary Fig. 15 show continuous rotation between different polarizations.

**Supplementary Section V: Electromechanical response at the PFs**

On the atomic scale, spontaneous polarization, $P_s$, can be calculated from the relative shift of the $B$-site atom with respect to the center of the oxygen octahedron using the equation

$$P_s = \frac{1}{V}\sum_i \delta_i Z_i \qquad \text{Eq. S4.}$$

where $Z_i$ is the effective charge of the $i$th atom with the ferroelectric displacement $\delta_i$, and $V$ is the unit-cell volume[19]. In the absence of $Z_i$ values, an empirical relation can be used to roughly estimate the $P_s$:

$$P_s = (258 \pm 9)\Delta z\ \mu C/cm^2 \qquad \text{Eq. S5.}$$

where $\Delta z$ is the displacement of B-site atom[18]. We can use this equation to measure the $P_s$ in the vicinity of PF. The $\delta_{NbO}$ values of 0.53 Å and 0.19 Å obtained from the DFT model near the PF (Fig. 3c), which are relatively higher than 0.06 Å and 0.16 Å obtained in the case of $NaNbO_3$ and $KNbO_3$ give a $P_s$ of 136.74±4.71 μC $cm^{-2}$ and 49.02±1.71 μC $cm^{-2}$, respectively[18]. The obtained value of 136.74±4.71 μC $cm^{-2}$ could have a large tolerance range due to the large difference as expected in the effective charge of Nb right next to the PF.

To develop the observed large electric-field-induced strain in PF-KNN, the electric field needs to induce large ionic displacements which can be of first-order (piezoelectric) or 2nd order (electrostrictive) in nature. In ferroelectrics, the electric-field-induced strain, $S_{33}$, caused by both piezoelectricity and electrostriction can be estimated by using the equation

$$S_{33} = 2Q_{33}\varepsilon_{33}P_sE_3 + Q_{33}\varepsilon_{33}^2E_3^2 \qquad \text{Eq. S6.}$$

where $Q_{33}$ and $\varepsilon_{33}$ are the electrostrictive coefficient and dielectric permittivity, respectively. Using $S_{33}$ = 0.016, $\varepsilon_{33}$ = 3 x $10^{-9}$ F $m^{-1}$, and $E_3$ = 83.3 kV $cm^{-1}$, the $P_s$ of 136.74 μC $cm^{-2}$ and 49.02 μC $cm^{-2}$ give us $Q_{33}$ of 0.25 $m^4$ $C^{-2}$ and 0.63 $m^4$ $C^{-2}$, respectively, compared to the $Q_{33}$ of 0.055 $m^4/C^2$ in $Pb(Mg_{1/3}Nb_{2/3})O_3$-x$PbTiO_3$ (PMN-xPT) relaxor single crystals[20].

An average value of the electrostrictive coefficient can also be obtained from the S-E curves using another equivalent form of $Q_{33}$ which is $M_{33}$ ($m^2$ $V^{-2}$) and can be calculated by using the following equation

$$S_{33} = M_{33} E_3^2 \qquad \text{Eq. S7.}$$

The effective $M_{33}$ of PF-KNN film can be calculated as 2.3 x $10^{-16}$ $m^2$ $V^{-2}$ at 83.3 kV $cm^{-1}$, which is almost twice compared to the effective $M_{33}$ of 1.18 x $10^{-16}$ $m^2$ $V^{-2}$ obtained in $Pb(Zn_{1/3}Nb_{2/3})O_3$–$xPbTiO_3$ (PZN-xPT) relaxor single crystals[21] at 120 kV $cm^{-1}$ and that of 0.6 – 1.1 x $10^{-16}$ $m^2$ $V^{-2}$ in (PMN-xPT) relaxor single crystals[20] at 80 kV $cm^{-1}$ as the best-known electrostrictors. The best-known $Q_{33}$ in KNN-based ceramics is only 0.051 $m^4$ $C^{-2}$ achieved by doping with different size and valence[22]. Hence, both from a local and average perspective, very large electrostrictive coefficients at the PFs would be required to develop such a large electromechanical response under an electric field. This suggests that other extrinsic factors are also involved in the giant electromechanical response other than the intrinsic electrostrictive response.

**Supplementary Section VI: Defect chemistry**

The formation of oxygen vacancy (OV) is one of the possible mechanisms for maintaining charge neutrality which is typically observed for $(K,Na)NbO_3$-based bulk ceramics. Theoretical calculations[23,24] and experiment[25] show that, in bulk alkali-niobate-based ceramics, the alkali vacancies are more likely to form compared to the OVs in oxygen-rich conditions. The formation of alkali vacancies results in the formation of holes:

$$Na_{Na/K}^{\times} \rightarrow V'_{Na/K} + h^{\bullet} \qquad \text{Eq. S8.}$$

Meanwhile, OVs are formed to keep the charge neutrality:

$$O_O^{\times} \rightarrow V_O^{\bullet\bullet} + 2e' \qquad \text{Eq. S9.}$$

However, in the case of thin-film growth with plasma, various other variables come into play, in addition to the relatively low processing temperature, such as the incoming flux of the atoms, ratio of atomic species available for phase nucleation, significant volatile loss, and high stress. Hence, the defect structure of thin films can vary greatly from their bulk counterparts even for similar compositions. Furthermore, the formation of Nb anti-site defects becomes energetically favorable in an alkali deficient atmosphere as observed in the present work. Hence, the excess Nb ions initially form anti-site defects at the faults i.e., $Nb_{Na}^{5+}$ or $Nb_{K}^{5+}$($Nb_{Na}^{4\bullet}$or $Nb_{K}^{4\bullet}$ in Kröger-Vink notation). For the sake of charge compensation, these anti-site $Nb_{Na/K}^{5+}$ ions can lower their valence state for example by raising their Fermi levels and becoming $Nb_{Na/K}^{4+}$. Such defects are also observed in $LiNbO_3$ ceramics where the formation of OVs is not favored[13]. The Nb valence state lowering, therefore, is considered the dominant charge compensation mechanism in our film. In fact, the planar fault can be considered as an excess $NbO_2$ plane (+1 charge) in the perfect lattice (ignoring the lattice shift). In this case, an excess uncompensated positive charge should be present at the faults, which can be compensated by the formation of oxygen interstitials $O_i^{''}$ rather than OVs, $V_O^{\bullet\bullet}$. However, we did not observe any interstitials as well, and thus it can only be compensated by lowering of Nb valence or the formation of more alkali vacancies.

Defects can also be characterized based on their respective activation energies. For example, the activation energies of single and doubly ionized oxygen vacancies are 0.7 eV and 1.2 eV respectively, which can be characterized by using impedance spectroscopy[26]. However, the activation energies calculated in this work using both the temperature-dependent conductivity and relaxation time lie between 0.3 – 0.4 eV which are significantly lower than that of OVs, hence, the OVs should not be the dominant defects present in the faulted KNN films. In case there was to be a high density of oxygen vacancies at the faults or in the vicinity, they would manifest as the decreased intensity of oxygen atomic columns in annular bright-field (ABF)

images such as in Fig. 2b) in the main text or the O K-edge intensity in the electron energy loss spectroscopy analysis (Fig 2h in the main text) at the faults. However, we did not observe such variations in our analysis. Based on these observations and analyses, we believe that the Nb valance state lowering should be the major charge compensation mechanism in the faulted KNN films.

**Supplementary Section VII: Measurement of $d^{*}_{33,f}$ and electric-field-induced strain using laser scanning vibrometer (LSV)**

LSV method measures both the magnitude as well as the phase of surface vibration for all the scanned points over a large area and offers comprehensive information on vibration modal shape. By examining the vibration modal shape, distorted or unexpected modality if any, including the artifacts such as from the peeling of electrode or film delamination, can be identified from the measurement. In contrast, single-point laser (doppler) vibrometer or double-beam laser interferometer (DBLI) measurements cannot give such information on vibration modality. In addition, LSV eliminates any inaccuracy caused by the misalignment of the double beams in DBLI. The piezoelectric measurements obtained using this method have been well validated by comparing the results from LSV and DBLI[27]. To further validate the reliability of the results, we performed LSV measurements at different voltages and frequencies on the control KNN sample (see Supplementary Fig. 2) which showed a strain = 0.05% and $d^{*}_{33,f}$ = 42.5 pm $V^{-1}$ (reported $d^{*}_{33,f}$ = 40 – 75 pm $V^{-1}$, see Supplementary Table 1).

**Supplementary Table 1.** Comparison of effective piezoelectric coefficients from this work and previously reported piezoelectric thin films.

| Composition | Substrate | Growth process | $d^*_{33,f}$ (pm $V^{-1}$) | Frequency (kHz) | Ref. |
|---|---|---|---|---|---|
| $(K_{0.26}Na_{0.74})_{0.52}NbO_{3-z}$ (PF-KNN) | $Nb:SrTiO_3$ | Sputtering | 1909 | 1 | This work |
| $Na_{0.86}NbO_{2.93}$ (NPR-NNO) | $Nb:SrTiO_3$ | Sputtering | 1098 | 1 | 1 |
| $Pb(Zr_{0.52}Ti_{0.48})O_3$ (PZT) | $LaNiO_3/Ca_2Nb_3O_{10}$/glass | PLD | 490 | 1 | 2 |
| $Pb(Zr_{0.52}Ti_{0.48})O_3$ (PZT) | $LaNiO_3/Pt/Ti/SiO_2/Si$ | PLD | 408 | 1 | 3 |
| $(Ba_{0.85}Ca_{0.15})(Ti_{0.9}Zr_{0.1})O_3$ (BCZT) | $Pt/Ti/SiO_2/Si$ | PLD | 300 | Not Specified | 4 |
| $0.95(K_{0.48}Na_{0.52})(Nb_{0.95}Sb_{0.05})O_3$-$0.05Bi_{0.5}(Na_{0.82}K_{0.18})_{0.5}ZrO_3$ (0.95KNNS-0.05BNKZ) | $Pt/Ti/SiO_2/Si$ | CSD | 250 | 1.5 | 5 |
| [001] $0.95(K_{0.48}Na_{0.52})(Nb_{0.95}Sb_{0.05})O_3$-$0.05Bi_{0.5}(Na_{0.82}K_{0.18})_{0.5}ZrO_3$ (0.95KNNS-0.05BNKZ) | $Pt/Ti/SiO_2/Si$ | CSD | 184 | 1.5 | 6 |
| $(Bi_{0.5}Na_{0.5})_{0.94}Ba_{0.06}TiO_3$+0.5 mol% $MnO_2$ (BNBT-$MnO_2$) | $SrRuO_3/SrTiO_3$ | PLD | 120 | Not Specified | 7 |
| $K_{0.5}Na_{0.5}NbO_3$ (KNN) | $Pt/Ti/SiO_2/Si$ | Sputtering | 74 | Not Specified | 8 |
| $K_{0.5}Na_{0.5}NbO_3$ | $Pt/Ti/SiO_2/Si$ | Sputtering | 65.4 | Not Specified | 9 |
| Mn-doped $(K_{0.44}Na_{0.52}Li_{0.04})(Nb_{0.84}Ta_{0.1}Sb_{0.06})O_3$ | $SrRuO_3/SrTiO_3$ | PLD | 45 | 17 | 10 |
| $K_{0.5}Na_{0.5}NbO_3$ | $Pt/Ti/SiO_2/Si$ | CSD | 40 | 17 | 11 |

PLD: Pulsed Laser Deposition, CSD: Chemical Solution Deposition. Ref. [1]Science 369, 292 (2020) [2]Sci. Rep. 7, 12915 (2017) [3]ACS Appl. Mater. Interfaces 9, 9849 (2017) [4]J. Appl. Phys. 125, 244103 (2019) [5]Adv. Electron. Mater. 5, 1800691 (2019) [6]Adv. Electron. Mater. 3, 1700033 (2017) [7]J. Am. Ceram. Soc. 99, 2347 (2016) [8]J. Am. Ceram. Soc. 98, 119 (2015) [9]J. Am. Ceram. Soc. 94, 1970 (2011) [10]J. Phys. D Appl. Phys. 43, 025405 (2010) [11]J. Phys. D Appl. Phys. 42, 215304 (2009)

**Supplementary Table 2.** Comparison of maximum strain from this work and previously reported piezoelectric and electrostrictive bulk ceramics, single crystals, and thin films.

| Material Composition | Polycrystalline/Single Crystal | Applied voltage (kV $cm^{-1}$) | Strain (%) | Ref. |
|---|---|---|---|---|
| $(K_{0.26}Na_{0.74})_{0.52}NbO_{3-z}$ (PF-KNN) | Single crystal with faults (Thin Film) | 83.3 | 5.6 | This work |
| $Pb(Zn_{1/3}Nb_{2/3})O_3$ -0.08$PbTiO_3$ (0.92PZN-0.08PT) | Single Crystal (Bulk) | 120 | 1.7 | [1] |
| $0.55Bi_{0.7}La_{0.3}FeO_3$–$0.45PbTiO_3$ (0.55BLFO-0.45PT) | Polycrystalline (Bulk) | 80 | 1.3 | [2] |
| $Pb(Zr_{0.52}Ti_{0.48})O_3$ (PZT) | Polycrystalline (Thin Film) | 200 | 1.0 | [3] |
| $K_{0.43}Na_{0.57}NbO_3$ (KNN43) | Single Crystal (Bulk) | 10 | 0.9 | [4] |
| $(Bi_{0.5}Na_{0.5})_{0.94}Ba_{0.06}TiO_3$+0.5 mol%$MnO_2$ (BNBT-$MnO_2$) | Single Crystal (Thin Film) | 600 | 0.875 | [5] |
| $Pb(Mg_{1/3}Nb_{2/3})O_3$ – $0.28PbTiO_3$ (0.72PMN-0.28PT) | Single crystal (Bulk) | 80 | 0.39 | [6] |
| $0.96(K_{0.5}Na_{0.5})(Nb_{0.965}Sb_{0.035})O_3$–$0.01SrZrO_3$ $0.03Bi_{0.5}Na_{0.5}ZrO_3$ (0.96KNNS-0.01SZ-0.03BNZ) | Polycrystalline (Bulk) | 40 | 0.22 | [7] |
| 2% Fe doped $Ba(Zr_{0.2}Ti_{0.8})O_3$-$0.5(Ba_{0.7}Ca_{0.3})TiO_3$ (Fe-BCZT) | Polycrystalline (Bulk) | 30 | 0.14 | [8] |
| $Bi_{0.5}Na_{0.5}TiO_3$–$BaTiO_3$–$KNbO_3$ (BNT-BT-KN) | Polycrystalline (Bulk) | 40 | 0.11 | [9] |
| $K_{0.5}Na_{0.5}NbO_3$-$SrTiO_3$ (KNN-ST) | Polycrystalline (Bulk) | 20 | 0.006 | [10] |

Ref.: [1]J. Appl. Phys. 82, 1804 (1997) [2]Nat. Mater. 17, 427 (2018) [3]Sci. Rep. 7, 12915 (2017) [4]Sci Adv 6, eaay5979 (2020) [5]J. Am. Ceram. Soc. 99, 2347 (2016) [6]Appl. Phys. Lett. 102, 152910 (2013) [7]J. Mater. Chem. 7, 2037 (2019) [8]Appl. Phys. Lett. 105, 232903 (2014) [9]J. Appl. Phys. 114, 027004 (2013) [10]J. Appl. Phys. 98, 024113 (2005)

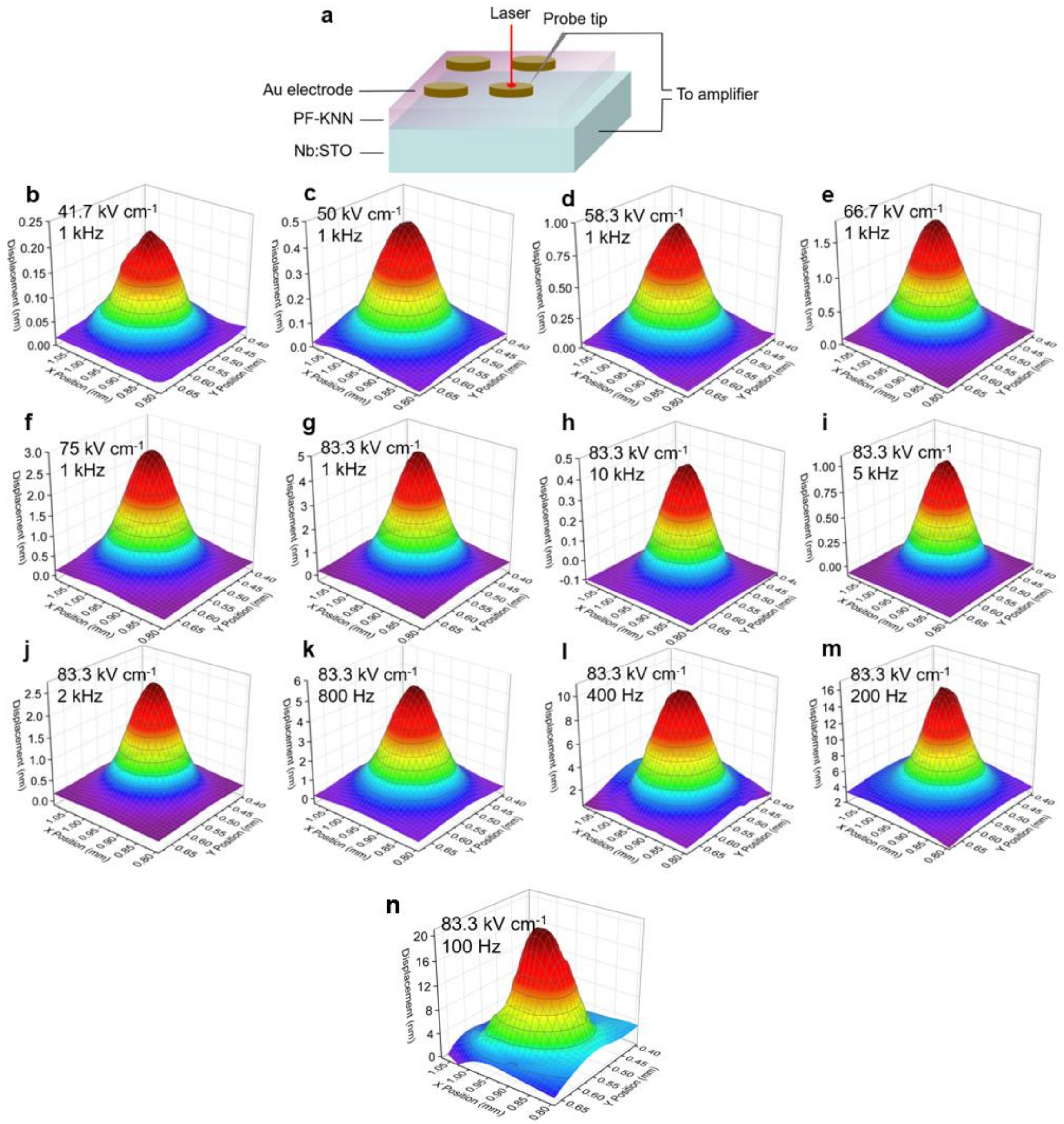


**Supplementary Fig. 1 Effective piezoelectric properties of PF-KNN**. **a,** Schematic of thin-film configuration for LSV measurements. 3D profiles of the surface displacement for PF-KNN thin-film under electric fields of **b,** 41.7 kV $cm^{-1}$ (1.25 V) **c,** 50 kV $cm^{-1}$ (1.5V), **d,** 58.3 kV $cm^{-1}$ (1.75V), **e,** 66.7 kV $cm^{-1}$ (2 V), **f,** 75 kV $cm^{-1}$ (2.25 V), and **g,** 83.3 kV $cm^{-1}$ (2.5 V) measured at 1 kHz. 3D profiles of the surface displacement measured at the frequencies of **h,** 10 kHz, **i,** 5 kHz, **j,** 2 kHz, **k,** 800 Hz, **l,** 400 Hz, **m,** 200 Hz and **n,** 100 Hz under an electric field of 83.3 kV $cm^{-1}$

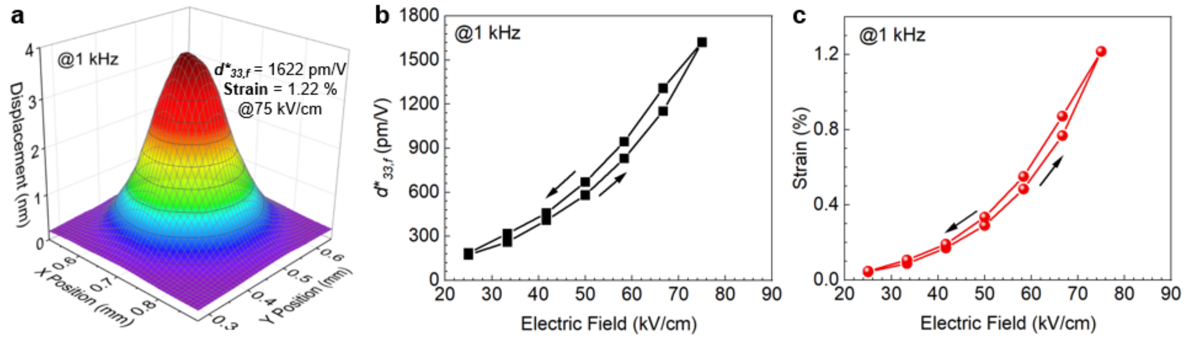


**Supplementary Fig. 2 Reversibility of electric-field-induced strain in PF-KNN film. a,** 3D profile of the surface displacement for PF-KNN thin-film measured under an electric field of 75 kV $cm^{-1}$. Variation in **b,** $d^*_{33,f,}$ and **c,** electric-field-induced strain in PF-KNN film with the applied electric field measured at 1 kHz frequency for both increasing and decreasing fields.

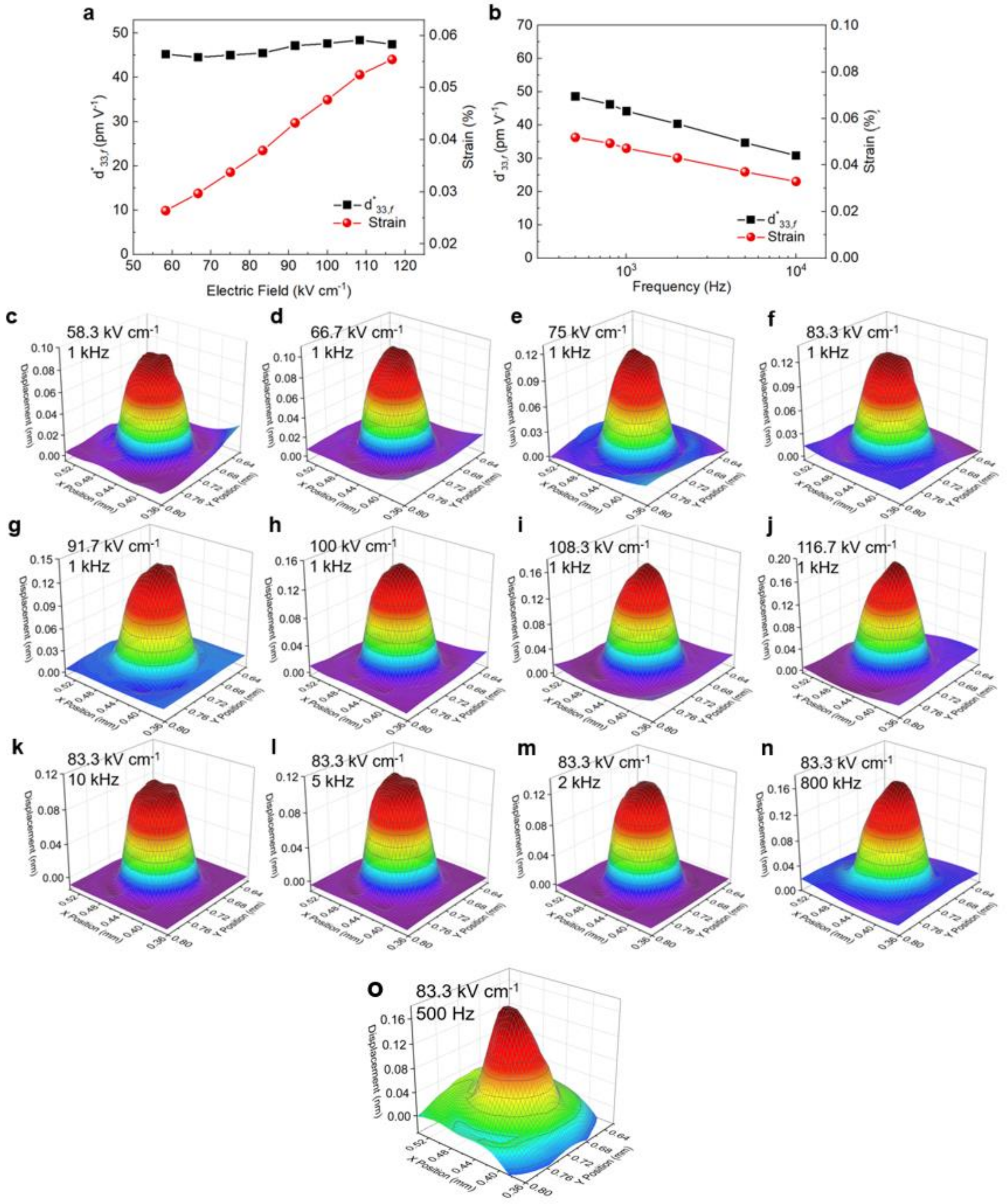


**Supplementary Fig. 3 Effective piezoelectric properties for the stoichiometric KNN thin film.** The variation of $d^*_{33,f}$ and the electric field-induced strain with **a,** the applied electric field, measured at 1 kHz, and **b,** the frequency, measured at the applied field of 83.3 kV $cm^{-1}$ for KNN thin film. 3D profiles of the surface displacement for under electric fields of **c,** 58.3 kV $cm^{-1}$, **d,** 66.7 kV $cm^{-1}$, **e,** 75 kV $cm^{-1}$, **f,** 83.3 kV $cm^{-1}$, **g,** 91.7 kV $cm^{-1}$, **h,** 100 kV $cm^{-1}$, **i,** 108.3 kV $cm^{-1}$, and **j,** 116.7 kV $cm^{-1}$ measured at 1 kHz frequency. 3D profiles of the surface displacement measured at the frequencies of **k,** 10 kHz, **l,** 5 kHz, **m,** 2 kHz, **n,** 800 Hz, and **o,** 500 Hz.

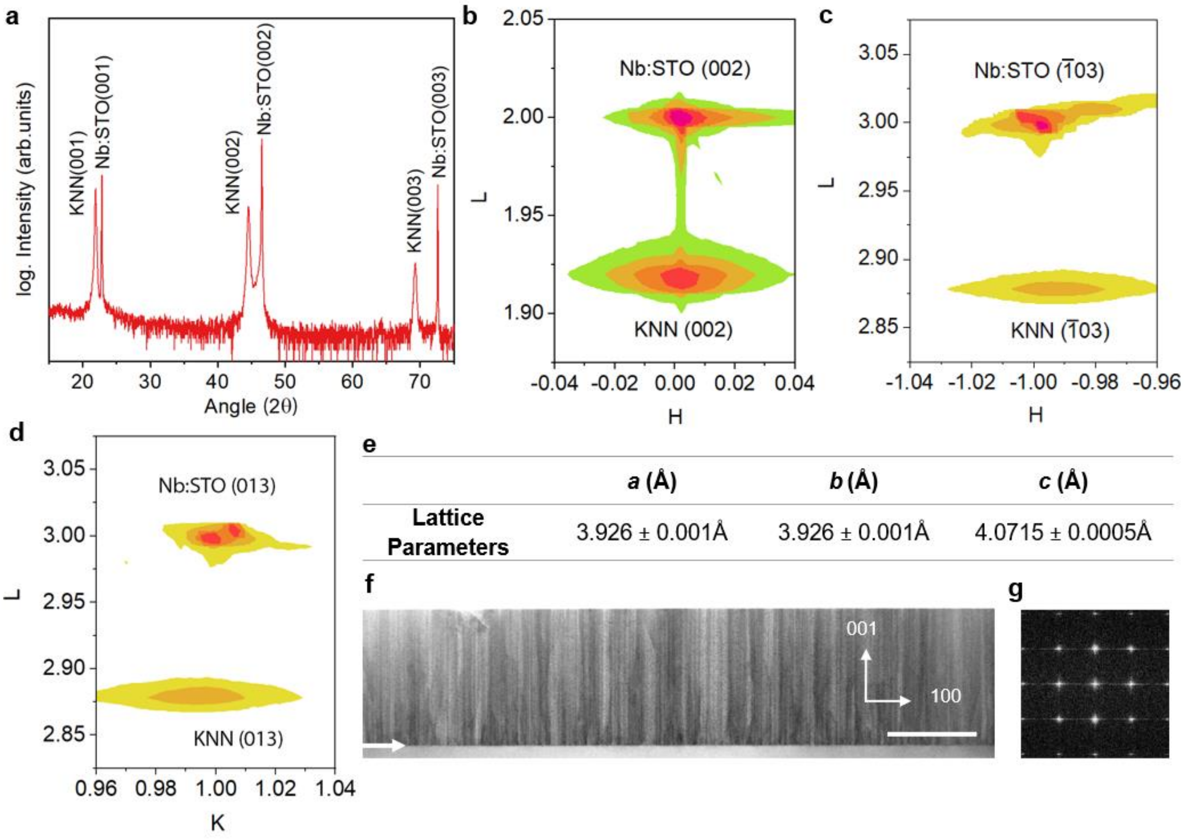


| | *a* (Å) | *b* (Å) | *c* (Å) |
|---|---|---|---|
| **Lattice Parameters** | 3.926 ± 0.001Å | 3.926 ± 0.001Å | 4.0715 ± 0.0005Å |

**Supplementary Fig. 4 Structural details for PF-KNN thin film. a,** Out-of-plane profile and RSMs around **b,** (002), **c,** $(\bar{1}03)$ and **d,** (013) planes. **e,** The in-plane and out-of-plane lattice parameters of PF-KNN thin film obtained from HR-XRD. **f,** A low magnification ABF image of thin-film cross-section. The arrow at the bottom points towards the film-substrate interface. The contrast variation is caused by the local elastic strain at the PFs and it is evident that the faults initiate from the film-substrate interface and extend to the film surface. The scale bar equals 200 nm. **g,** Fast Fourier transform (FFT) image of the thin-film region in (f) showing single-crystalline structure. The horizontal streaking is caused by the presence of planar faults.

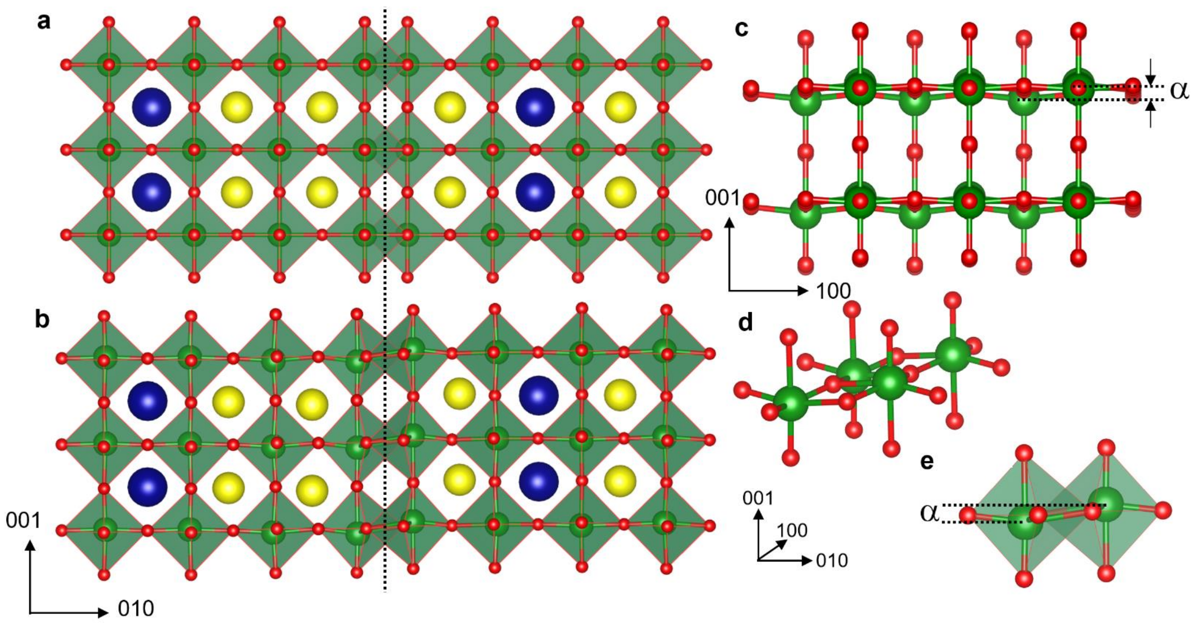


**Supplementary Fig. 5 Density functional theory (DFT) simulated structures.** Comparison of **a,** a perfect $a/2[\overline{1}\overline{1}0](100)$ type stacking fault and **b,** a distorted $a/2[\overline{1}\overline{1}0](100)$ type PF observed in PF-KNN thin film projected along [100] direction. The dotted line shows the positions of the fault. Our DFT calculations show that the distorted PF structure is more stable than the perfect stacking fault with an energy difference of $\Delta E = E_{DPF} - E_{PSF}$ = -31.54 meV per f.u ($E_{DPF}$ is the energy of distorted PF, and $E_{PSF}$ is the energy of perfect stacking fault). Blue spheres (K), yellow spheres (Na), green spheres (Nb), and red spheres (O) show representative atomic columns. **c,** Projection along [010] direction highlighting the vertical distortion $\alpha$. **d,** Three-dimensional view of the Nb-O bonds at the PF. **e,** Vertical displacement ($\alpha$) of Nb-O octahedral complexes relative to each other, at the PF. These defects are different from the well-known Ruddlesden-Popper (RP) type faults which are characterized as an extra AO layer (for $ABO_3$ type materials) at the fault interface instead of $BO_2$ and without the sharing of oxygen octahedra. Furthermore, the RP faults are $a/2[111](100)$ type in nature indicating exchange between A and B atomic sites across the fault. However, such is not the case in these novel defects in this work.

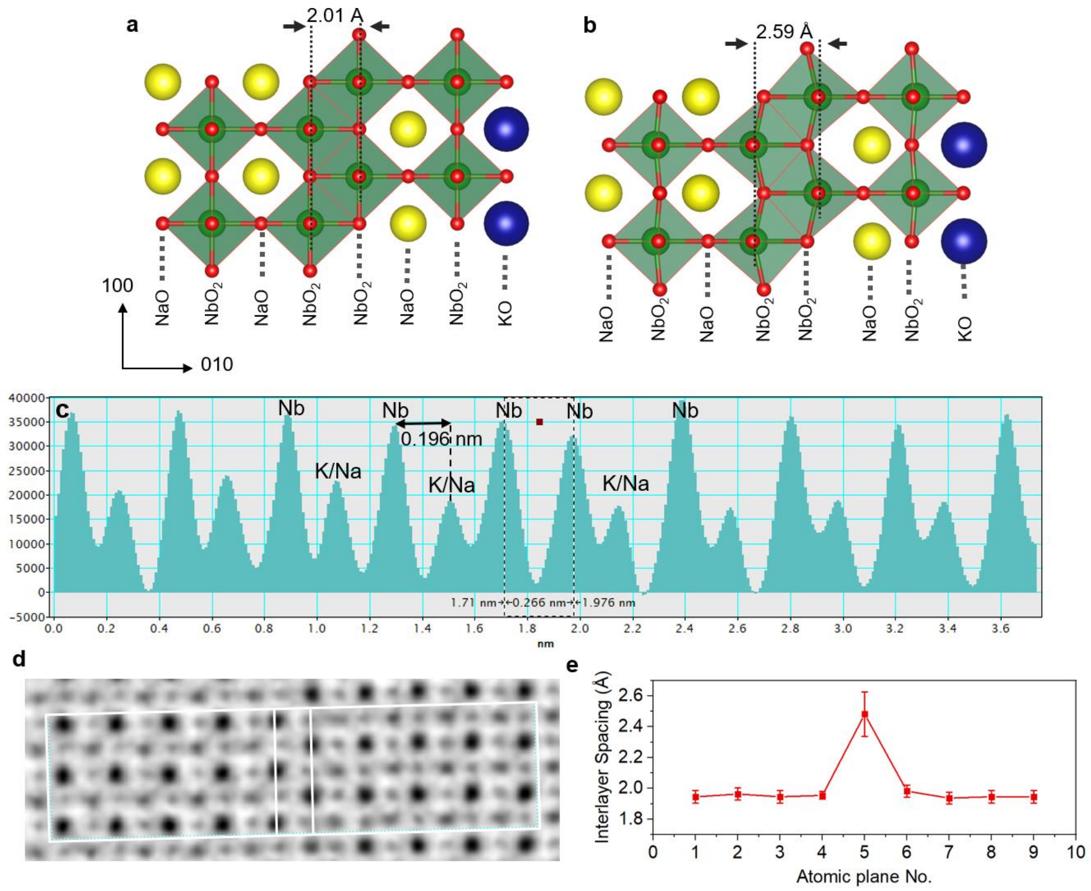


**Supplementary Fig. 6 Measurement of $NbO_2$-$NbO_2$ interplanar distance at the PF.** Comparison of **a,** a perfect $a/2[\overline{1}\overline{1}0](100)$ type stacking fault and **b,** a distorted $a/2[\overline{1}\overline{1}0](100)$ type PF observed in the PF-KNN thin film projected along [001] direction. The distance between the adjacent $NbO_2$-$NbO_2$ planes at the PF increases from 2.01 Å to 2.59 Å in the distorted PF as calculated by the DFT simulations. The structure of the PF in (b) matches well with the STEM images obtained from the PF-KNN thin film surface (Fig. 2b in the main text). **c,** Intensity profile obtained from the rectangular region in **d,** which is an ABF STEM image of thin-film plan-view with a PF in the center. The $NbO_2$-$NbO_2$ distance at the PF is 0.266 nm (2.66 Å) whereas the interplanar distance (between adjacent $NbO_2$ and KO/NaO planes) is 0.196 nm (1.96 Å). **e,** Variation of interlayer distance as approaching a PF (at atomic plane No. 5). Error bars represent standard deviation after averaging distances obtained from five different regions containing a PF. The interplanar distances were obtained by measuring the distance between $NbO_2$ layers which were halved (except for the $NbO_2$-$NbO_2$ distance at the PF) and plotted in (e) to avoid the variation due to polar displacements of Nb with respect to KO/NaO layers.

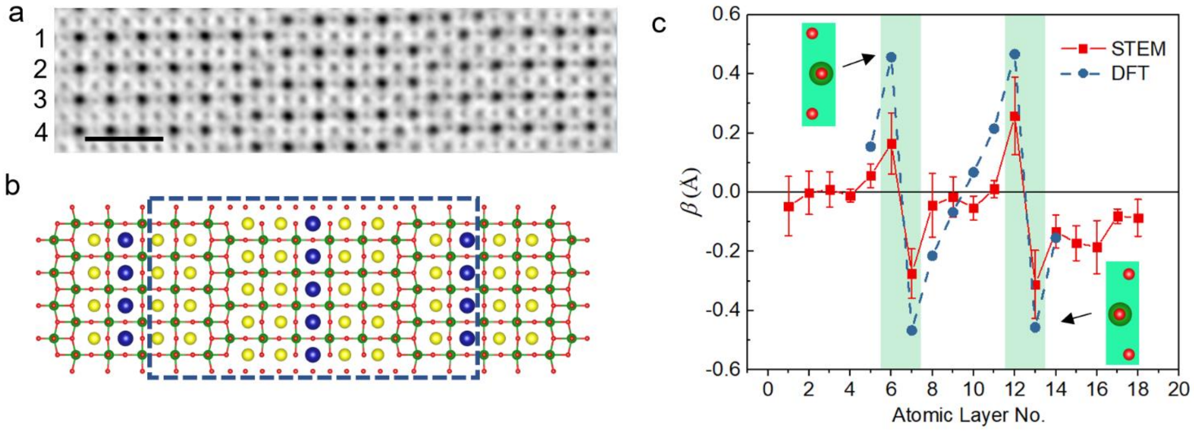


**Supplementary Fig. 7 The rumpling of oxygen atomic planes surrounding the PFs**. **a,** ABF image of thin-film plan-view containing two parallel PFs. Scale bar equals 1 nm. **b,** The corresponding structural model obtained from DFT. Yellow spheres represent the Na atoms, blue spheres represent the K atoms, green spheres represent the Nb atoms and red spheres represent the O atoms. **c,** Average β values obtained from (a) and (b) by subtracting the horizontal shift of the oxygen atom from the Nb atoms, repeated for all the vertical $NbO_2$ layers. The error bars represent the standard deviation resulting from averaging the β values obtained from different horizontal planes. Rumpling on the right side of the $NbO_2$ plane is arbitrarily set as positive $\beta$. Highlighted regions indicate the position of PFs.

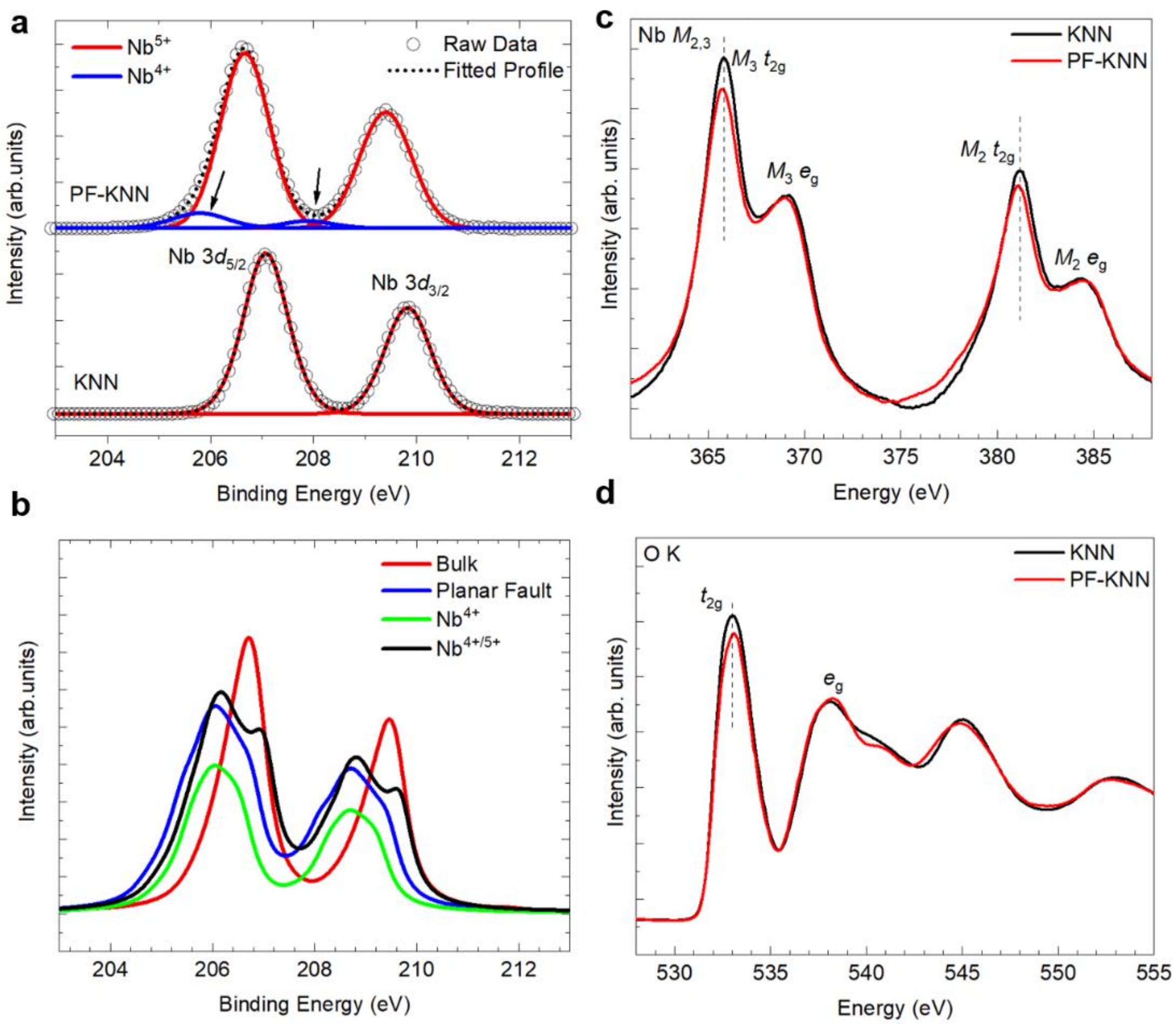


**Supplementary Fig. 8 Spectroscopic analysis of PF-KNN film a,** XPS spectra of Nb 3*d* edges for the KNN and PF-KNN thin films. Additional peaks centered at 205.79 eV and 207.89 eV, respectively, visible in the spectrum of PF-KNN film (highlighted by arrows) compared to that of the KNN thin film can be attributed to $Nb^{4+}$ ions. **b,** XPS spectra of Nb 3*d* from the first-principles calculation. The simulation was conducted by removing one electron from one Nb atom at the planar interface, to create an $Nb^{4+}$ ion, and the electron was inserted at the planar interface after which the electronic structure was reoptimized and the XPS was conducted. The obtained results show the XPS peak for bulk Nb atoms (having 5+ valence) shown by the red curve. The peak at the shoulder (206 eV) comes from the $Nb^{4+}$ atom shown by the green curve. The XPS peaks for Nb atoms at the planar interface, shown by the blue curve, are in the same position as $Nb^{4+}$ which confirms the presence of $Nb^{4+}$ ions at the PF interface. The XPS spectra for the Nb atom next to the interface, shown by the black curve, split into two peaks with the valence of +4 and 5+. The theoretical simulation therefore well supports that the valence of Nb atoms at the interface is lower than 5+. **c,** Nb $M_{2,3}$, and **d,** O K-edge. The Nb M-edge corresponds to the transition from 3*p* to 4*d* level, where $M_3$ is attributed to the $3p_{3/2}$ and $M_2$ to the $3p_{1/2}$ states. Since Nb atoms are surrounded by O atoms forming oxygen octahedra, the 4d orbital splits into $t_{2g}$ and $e_g$ components, where $t_{2g}$ lies at relatively lower energy. Hence, the two Nb M-edge peaks split into two each, totaling four peaks as labeled in a. Two differences can be observed in the $M_{2,3}$ edge obtained in PF KNN from KNN. First, the PF-KNN peak is shifted slightly towards lower energy compared to that of KNN film. Secondly, the peak intensity ratio of both $M_3$ $t_{2g}$ to $M_3$ $e_g$ and $M_2$ $t_{2g}$ to $M_2$ $e_g$ peaks is decreased in the case of PF-KNN compared to that of KNN. Both these signs indicate the partial filling of the Nb 4*d* level of PF-KNN film resulting in a lower valence state than 5+ of Nb in the KNN film. The O K-edge indicates the hybridization between Nb 4*d* and O 2*p* orbitals. Here the decrease in the peak intensity ratio of $t_{2g}$ to $e_g$ also indicates the lowering of Nb valence in PF-KNN film compared to KNN film, consistent with the observed in O K-edge obtained via EELS.

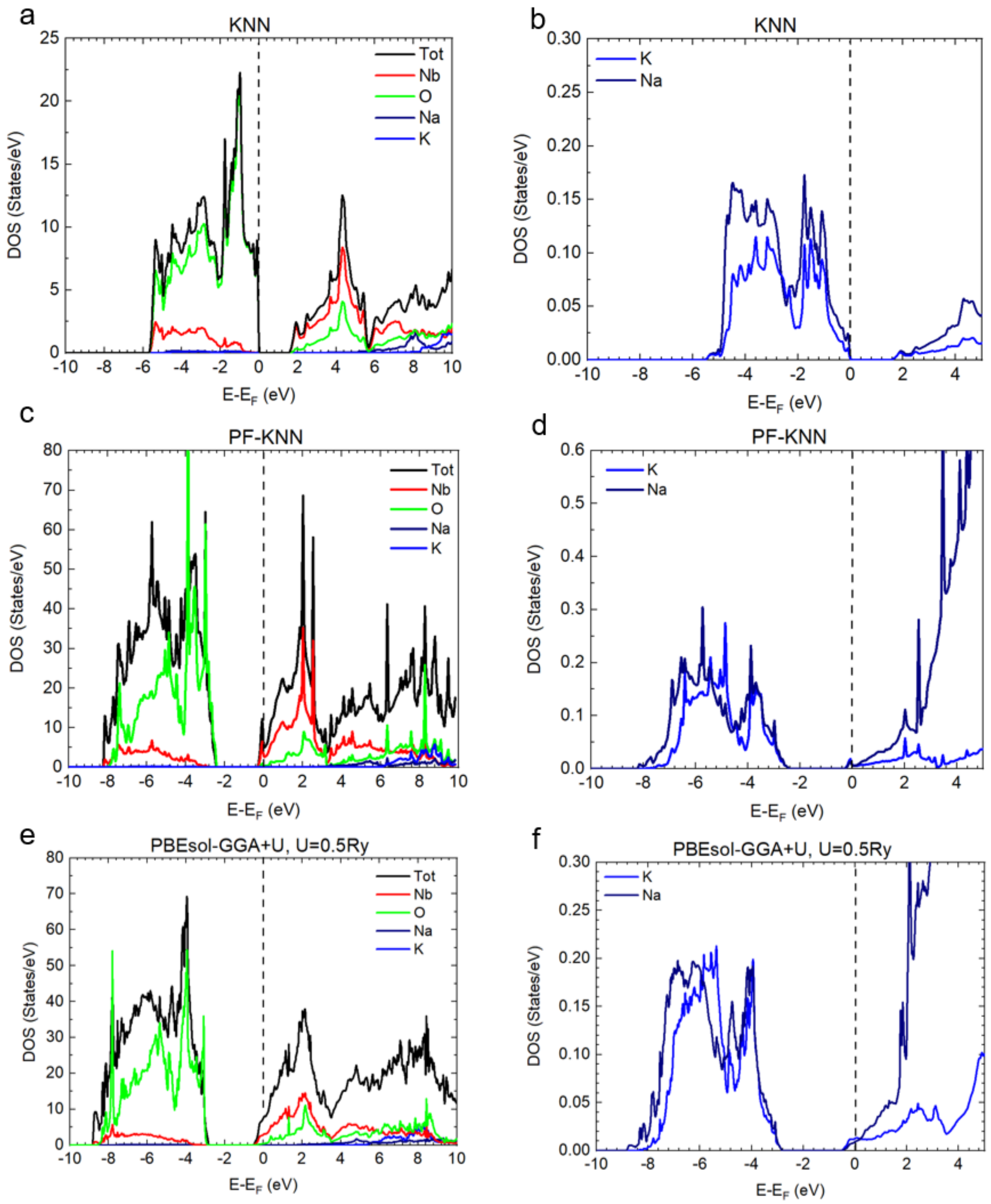


**Supplementary Fig. 9 Density of states (DOS) calculated** for **a,** KNN and **c,** PF-KNN film. **b,** and **d,** show the enlarged view of (a) and (c) respectively for K and Na. The Fermi level is located at E=0 as highlighted by dotted lines in the plots. In KNN, the valence band is dominated by O-states with contributions from Nb, whereas the K- and Na-states show insignificant contributions. The conduction band, however, is dominated by Nb-states with some contribution from O. Based on the DOS given in (a), KNN shows a semiconducting behavior. With the addition of planar faults in KNN, the whole DOS is shifted to the lower energy compared to the pristine KNN structure as shown in (c). The Fermi level for PF-KNN is in the conduction band showing the n-type nature of the system due to the excess electrons at the PF. This further supports the lowering of the ionic charge of Nb at PFs due to charge redistribution. Overall, PF-KNN shows metallic behavior. **e,f** PBE-GGA+*U* calculations for PF-KNN structure with *U*=0.5Ry (6.8 eV). The results show that the electron correlation has no significant effect on the electronic properties of PF-KNN.

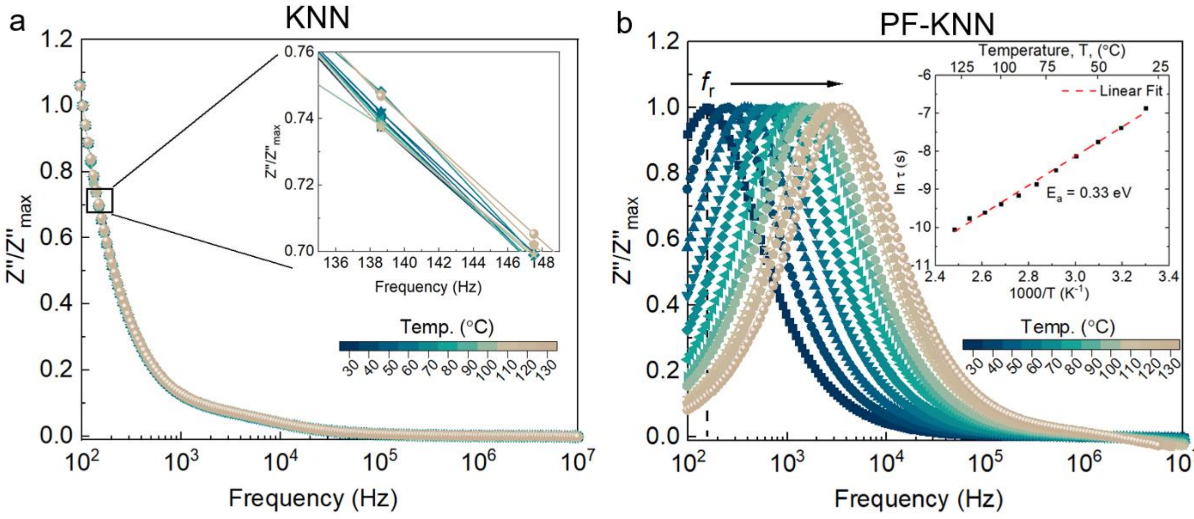


**Supplementary Fig. 10 AC impedance spectroscopy of KNN and PF-KNN film**. **a,** Variation of the normalized imaginary part of the complex impedance (Z") with the frequency measured for KNN film at the different temperatures. The inset shows a magnified view. **b,** Variation of the normalized Z" with the frequency measured at the different temperatures for PF-KNN film. $f_r$ represents the relaxation frequency of the charged defects. The inset shows the activation energy $E_a$ calculated from impedance relaxation peaks measured at different temperatures. Relaxation time $\tau$ is calculated by using the formula $\tau = (2\pi f_r)^{-1}$.

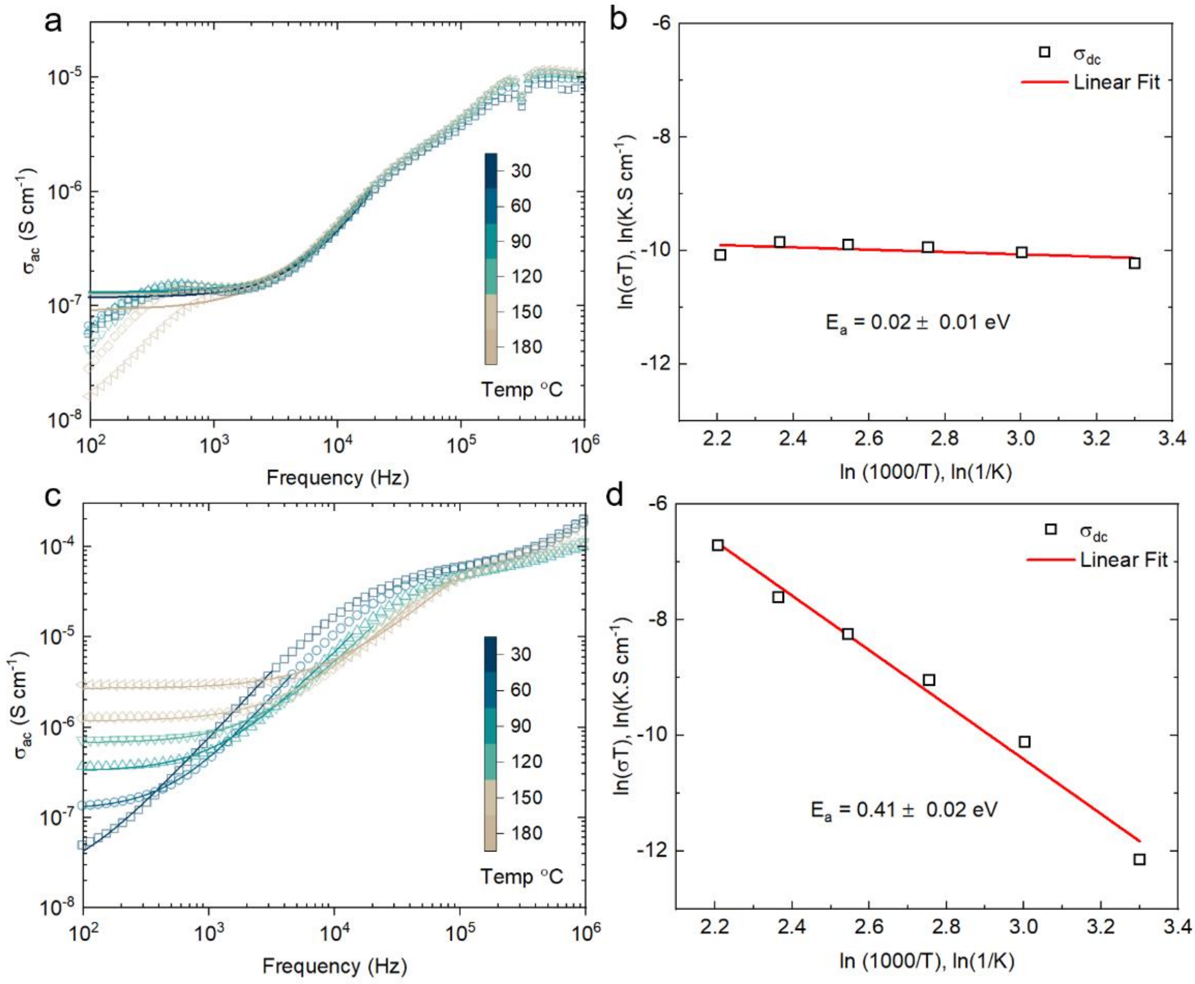


**Supplementary Fig. 11 AC conductivity analysis of KNN and PF-KNN films. a,** Variation of AC conductivity with the frequency measured at different temperatures for KNN film. Symbols represent raw data points and solid lines show fitting by Jonscher's power law. **b,** Calculation of activation energy from dc conductivity $\sigma_{dc}$ obtained from (a). **c,** Variation of AC conductivity with the frequency measured at different temperatures for PF-KNN film. Symbols represent raw data points and solid lines show fitting by Jonscher's power law. **d,** Calculation of activation energy from dc conductivity $\sigma_{dc}$ obtained from (c).

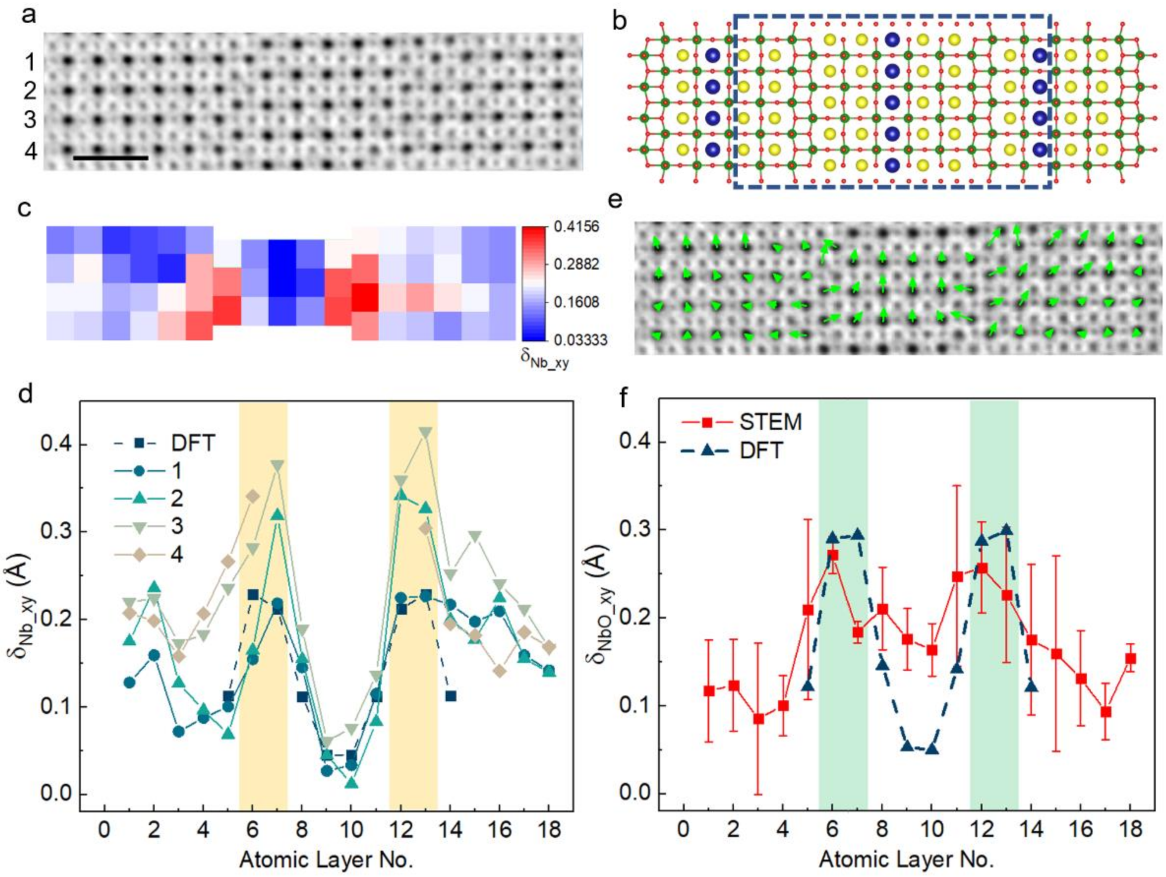


**Supplementary Fig. 12 Effect of PFs on the surrounding polarization. a,** ABF image of thin-film plan-view containing two parallel PFs. Scale bar equals 1 nm. **b,** The corresponding structural model obtained from DFT. Yellow spheres represent the Na atoms, blue spheres represent the K atoms, green spheres represent the Nb atoms and red spheres represent the O atoms. **c,** Magnitude of the in-plane shift of Nb atoms i.e. $\delta_{Nb\_xy}$ obtained from (a). One pixel represents a single unit cell. **d,** Magnitude of the in-plane shift of Nb atoms i.e., $\delta_{Nb\_xy}$ obtained from (a) and (b). The $\delta_{Nb\_xy}$ values were obtained from all the rows separately where 1 represents the top-most row of atomic cells and 4 shows the lowest one. Highlighted regions indicate the position of PFs **e,** The in-plane shift of Nb atoms with respect to the oxygen octahedra shown as a vector ($\delta_{NbO\_xy}$) map overlayed on (a). **f,** Magnitude of $\delta_{NbO\_xy}$ obtained from (e). Highlighted regions indicate the position of PFs. The error bars represent the standard deviation resulting from averaging the $\delta_{NbO\_xy}$ values obtained from different horizontal planes

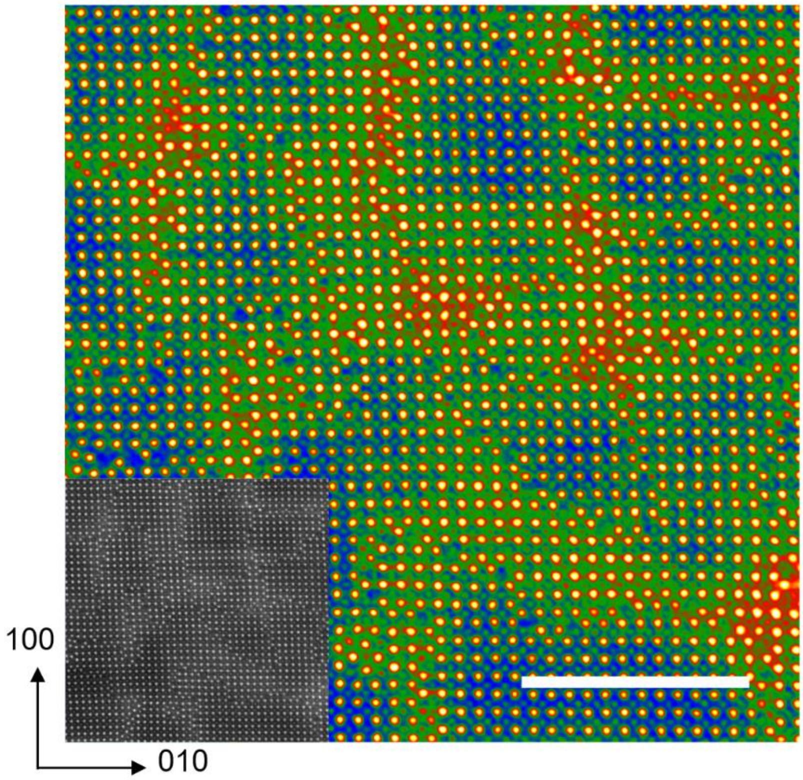


**Supplementary Fig. 13 Low angle annular dark-field (LAADF) image of thin-film plan-view** (false-colored). LAADF imaging mode is very sensitive to stress variations which are visible as bright regions in the vicinity of PFs. Inset is the original micrograph. Scale bar equals 5 nm.

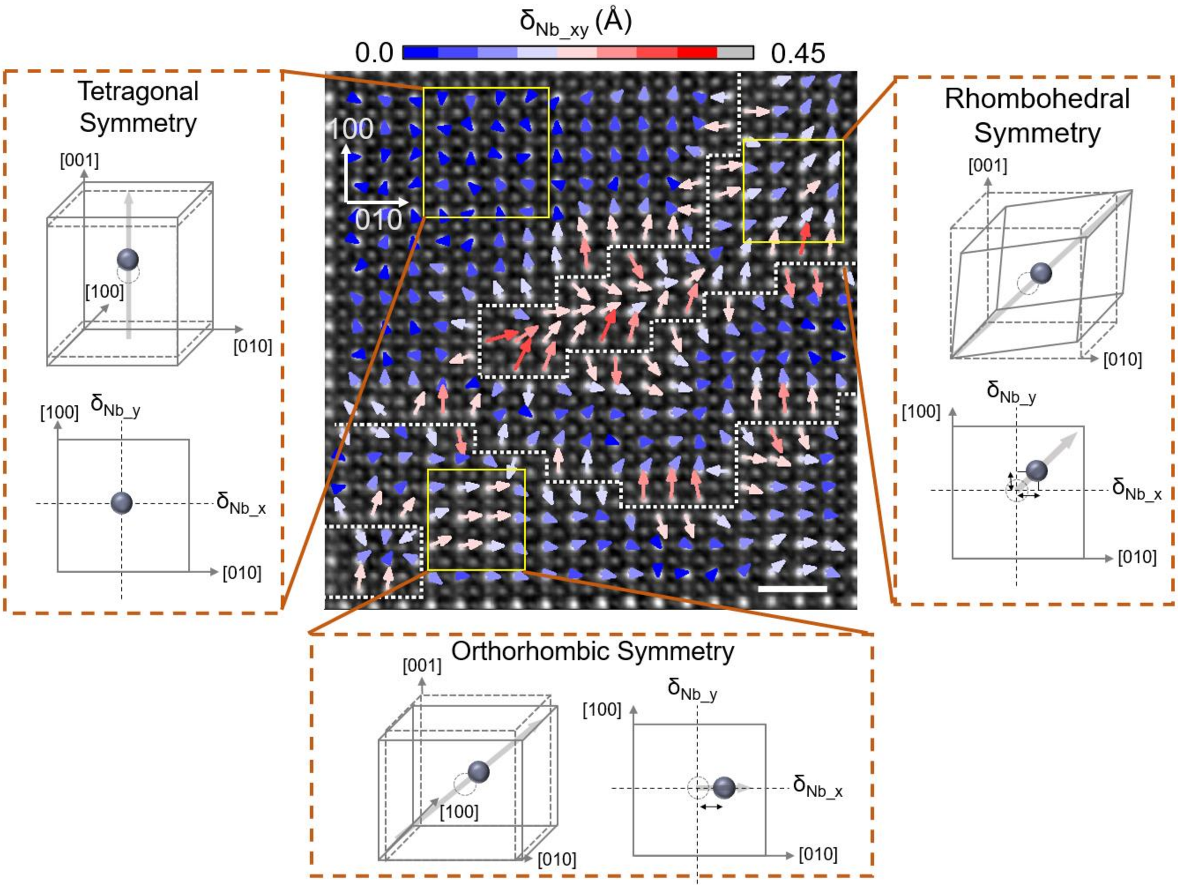


**Supplementary Fig. 14 In-plane polarization vector $\boldsymbol{\delta_{Nb\_xy}}$ map** overlayed on inverted ABF image of thin-film plan-view where white dotted lines show the planar faults. Scale bar equals 1 nm. A tetragonal phase is observed in the matrix perovskite KNN phase whereas low symmetry rhombohedral and orthorhombic phases are observed in the vicinity of the PFs with the possible presence of bridging monoclinic phases to facilitate polarization rotation between the PFs and the matrix.

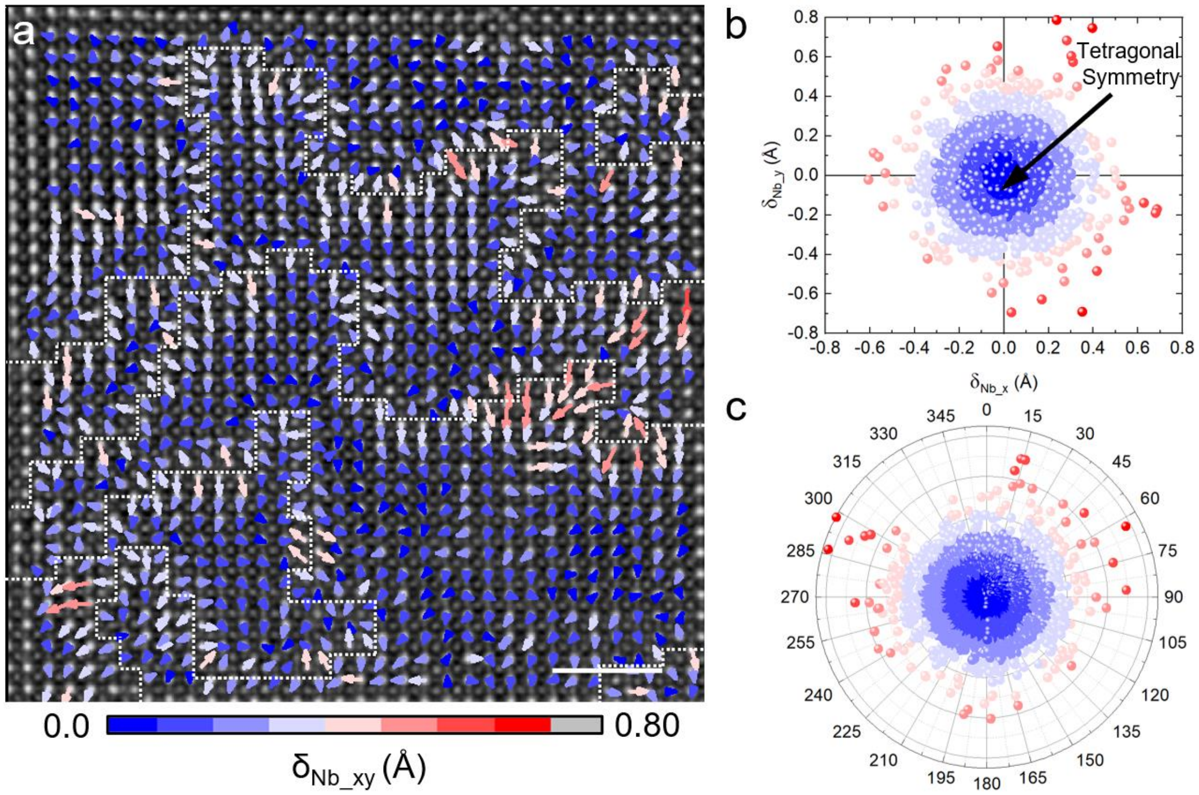


**Supplementary Fig. 15 Local polarization analysis and domain structure of PF-KNN thin film. a,** In-plane polarization vector $\delta_{Nb\_xy}$ map overlayed on inverted ABF image of thin-film plan-view where white dotted lines show the planar faults. Scale bar equals 2 nm. **b,** and **c,** represent the distribution of polarization magnitude and angle, respectively.

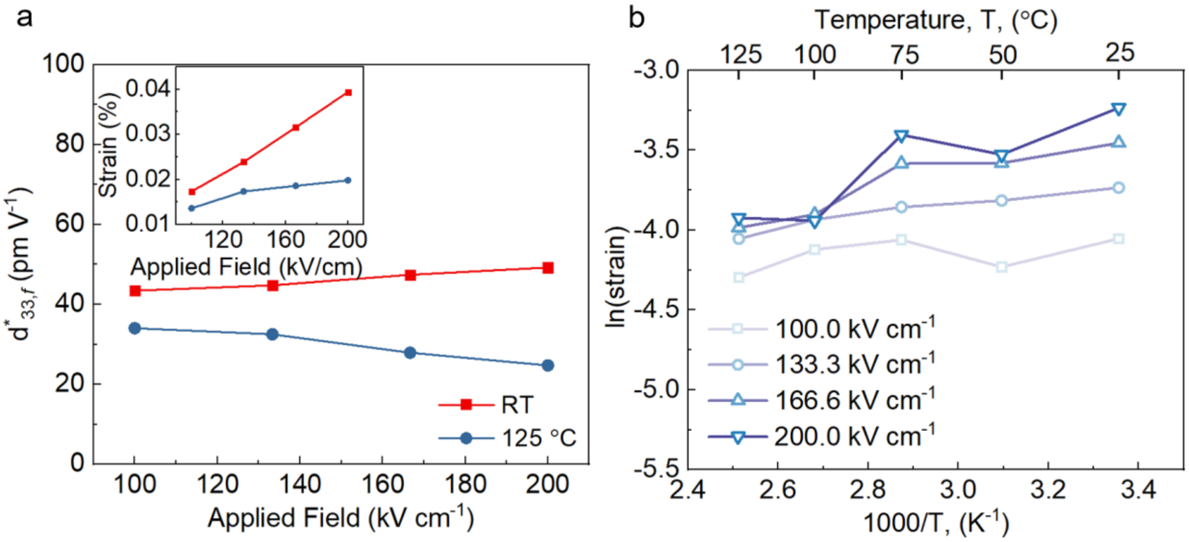


**Supplementary Fig. 16 High-temperature electromechanical characterization of KNN thin film a,** Variation in $d^{*}_{33,f}$ (and strain in the inset) measured at room temperature and 125˚C. **b,** Variation in the film strain with temperature under different external electric fields presented in Arrhenius formalism. All the piezoelectric measurements were done at 1 kHz.

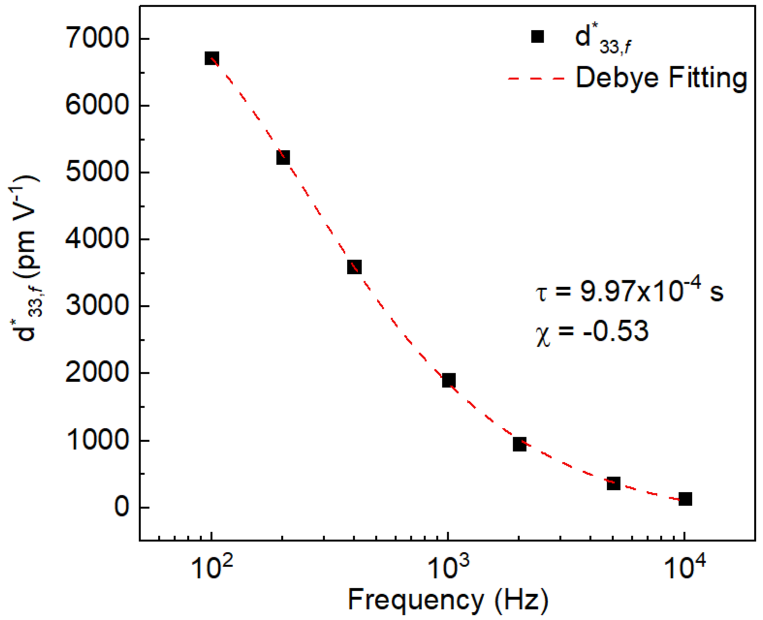


**Supplementary Fig. 17 Frequency dependence of $d^*_{33,f}$ for PF-KNN film**. The fitting of frequency-dependent electric field-induced strain with the non-ideal Debye equation $S(\omega) = \frac{S_S - S_\infty}{\sqrt{1+(\omega\tau)^{2+\chi}}} + S_\infty$, where $\omega = 2\pi f$, $S_s$ is the static (dc) value of strain, $S_\infty$ is the strain at infinite (high) frequency, $\tau$ is the relaxation time and $\chi$ is the non-ideality factor

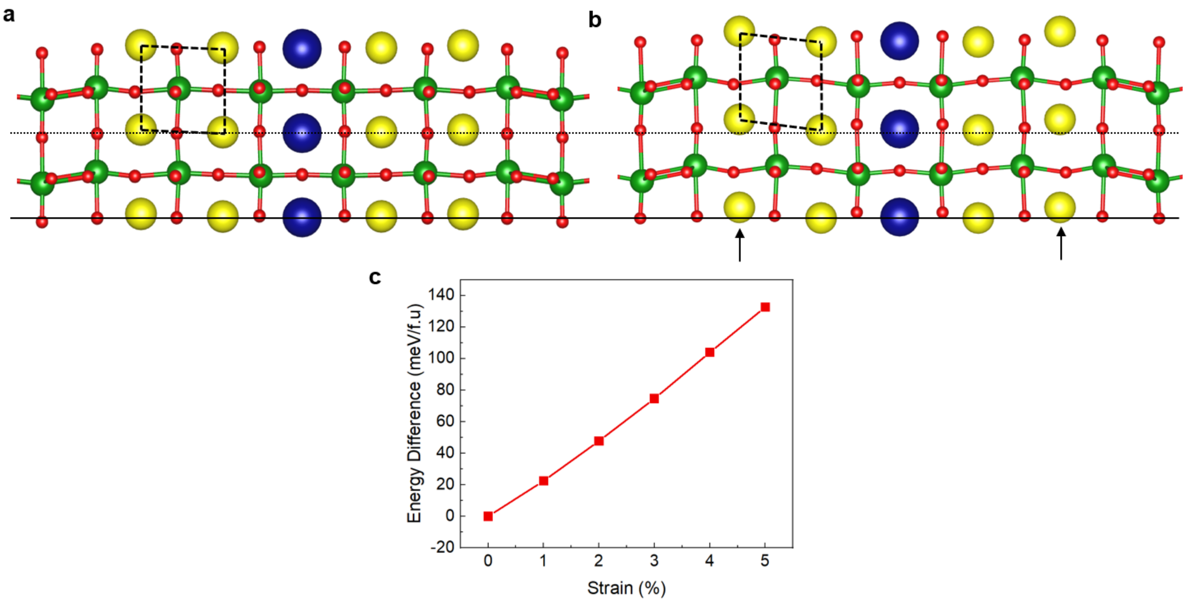


**Supplementary Fig. 18 DFT simulated structure of a KNN supercell consisting of 4 cells of nominal perovskite structure constrained by two planar faults at both sides,** with an out-of-plane lattice parameter *c* of **a,** 4.05 Å and **b,** 4.25 Å. The atomic cells adjacent to the PFs (pointed out by arrows) deform more than the atomic cells in the middle after ~5% out-of-plane straining of the supercell. The structure shows considerable lattice shearing specifically near the planar faults along with the large out-of-plane deformation of oxygen octahedra. **c,** Energy difference of PF-KNN structure with different out-of-plane lattice parameter *c* compared with that of 4.05 Å (for which the energy difference is zero). ‘Strain’ here indicates the percentage increase in *c* compared to 4.05 Å.

## Supplementary References


1. Liu, H. *et al.* Giant piezoelectricity in oxide thin films with nanopillar structure. *Science* **369**, 292–297 (2020).
2. Cook, W. R., Jaffe, H. & Jaffe, B. *Piezoelectric ceramics*. (Academic Press, 1971).
3. Ahtee, M. & Glazer, A. M. Lattice parameters and tilted octahedra in sodium–potassium niobate solid solutions. *Acta Crystallogr. A* **32**, 434–446 (1976).
4. Tennery, V. J. & Hang, K. W. Thermal and X‐Ray Diffraction Studies of the NaNbO3–KNbO3 System. *J. Appl. Phys.* **39**, 4749–4753 (1968).
5. Levin, I. *et al.* Coupling of emergent octahedral rotations to polarization in (K,Na)NbO3 ferroelectrics. *Sci. Rep.* **7**, 15620 (2017).
6. Petkov, V., Kim, J.-W., Shastri, S., Gupta, S. & Priya, S. Geometrical frustration and piezoelectric response in oxide ferroics. *Phys. Rev. Materials* **4**, 014405 (2020).
7. Wu, L., Zhang, J. L., Wang, C. L. & Li, J. C. Influence of compositional ratio K/Na on physical properties in (KxNa1−x)NbO3 ceramics. *J. Appl. Phys.* **103**, 084116 (2008).
8. Bach, D., Störmer, H., Schneider, R., Gerthsen, D. & Verbeeck, J. EELS investigations of different niobium oxide phases. *Microsc. Microanal.* **12**, 416–423 (2006).
9. Orgel, L. E. *An introduction to transition-metal chemistry: ligand-field theory*. (Taylor & Francis, 1966).
10. Li, C. *et al.* Atomic scale characterization of point and extended defects in niobate thin films. *Ultramicroscopy* **203**, 82–87 (2019).
11. Linford, R. G. & Hackwood, S. Physical techniques for the study of solid electrolytes. *Chem. Rev.* **81**, 327–364 (1981).
12. Granzow, T. Polaron-mediated low-frequency dielectric anomaly in reduced $LiNbO_3$:Ti. *Appl. Phys. Lett.* **111**, 022903 (2017).

13. Schirmer, O. F., Imlau, M., Merschjann, C. & Schoke, B. Electron small polarons and bipolarons in $LiNbO_3$. *J. Phys. Condens. Matter* **21**, 123201 (2009).
14. Xu, T. *et al.* Electron engineering of metallic multiferroic polarons in epitaxial $BaTiO_3$. *npj Comput Mater* **5**, 1–7 (2019).
15. Mhaouech, I. & Guilbert, L. Temperature dependence of small polaron population decays in iron-doped lithium niobate by Monte Carlo simulations. *Solid State Sci.* **60**, 28–36 (2016).
16. Rafiq, M. A., Tkach, A., Costa, M. E. & Vilarinho, P. M. Defects and charge transport in Mn-doped $K_{0.5}Na_{0.5}NbO_3$ ceramics. *Phys. Chem. Chem. Phys.* **17**, 24403–24411 (2015).
17. Jonscher, A. K. The 'universal' dielectric response. *Nature* **267**, 673–679 (1977).
18. Abrahams, S. C., Kurtz, S. K. & Jamieson, P. B. Atomic displacement relationship to curie temperature and spontaneous polarization in displacive ferroelectrics. *Phys. Rev.* **172**, 551–553 (1968).
19. Zhong, W., King-Smith, R. D. & Vanderbilt, D. Giant LO-TO splittings in perovskite ferroelectrics. *Phys. Rev. Lett.* **72**, 3618–3621 (1994).
20. Li, F., Jin, L., Xu, Z., Wang, D. & Zhang, S. Electrostrictive effect in $Pb(Mg_{1/3}Nb_{2/3})O_3$-$xPbTiO_3$ crystals. *Appl. Phys. Lett.* **102**, 152910 (2013).
21. Park, S.-E. & Shrout, T. R. Ultrahigh strain and piezoelectric behavior in relaxor based ferroelectric single crystals. *J. Appl. Phys.* **82**, 1804–1811 (1997).
22. Wu, B. *et al.* Superior Electrostrictive Effect in Relaxor Potassium Sodium Niobate Based Ferroelectrics. *ACS Appl. Mater. Interfaces* **12**, 25050–25057 (2020).
23. Shigemi, A. & Wada, T. Enthalpy of Formation of Various Phases and Formation Energy of Point Defects in Perovskite-Type $NaNbO_3$ by First-Principles Calculation. *Jpn. J. Appl. Phys.* **43**, 6793 (2004).
24. Shigemi, A. & Wada, T. Evaluations of Phases and Vacancy Formation Energies in $KNbO_3$ by First-Principles Calculation. *Jpn. J. Appl. Phys.* **44**, 8048 (2005).

25. Lin, J. *et al.* Reversible modulation of photoenergy in Sm-doped $(K_{0.5}Na_{0.5})NbO_3$ transparent ceramics via photochromic behavior. *J. Mater. Chem. A Mater. Energy Sustain.* **7**, 19374–19384 (2019).

26. Li, R., Tan, B., Zheng, T. & Wu, J. Defect-driven conductivity behavior in lead-free KNN-based ceramics. *J. Appl. Phys.* **127**, 114103 (2020).

27. Herdier, R., Jenkins, D., Dogheche, E., Rèmiens, D. & Sulc, M. Laser Doppler vibrometry for evaluating the piezoelectric coefficient $d_{33}$ on thin film. *Review of Scientific Instruments* vol. 77 093905 (2006).
