# Supplementary material for: Origin of giant electric-field-induced strain in faulted alkali niobate films
Source: Nat Commun. 2022 Jul 7;13:3922. doi: 10.1038/s41467-022-31630-8 (PMC9262982; doi:10.1038/s41467-022-31630-8)
Supplement: Supplementary file 4 — Lasing Reporting Summary [file 41467_2022_31630_MOESM4_ESM.pdf]

nature research

Corresponding author(s): Kui Yao, Stephen J Pennycook, John Wang

Lasing Reporting Summary

Nature Research wishes to improve the reproducibility of the work that we publish. This form is intended for publication with all accepted papers reporting claims of lasing and provides structure for consistency and transparency in reporting. Some list items might not apply to an individual manuscript, but all fields must be completed for clarity.

For further information on Nature Research policies, including our data availability policy, see Authors & Referees.

ü Experimental design

Please check: are the following details reported in the manuscript?

1. Threshold

Plots of device output power versus pump power over a wide range of values indicating a clear threshold

☐ Yes ☒ No — Lasing experiments are not involved in this work.

2. Linewidth narrowing

Plots of spectral power density for the emission at pump powers below, around, and above the lasing threshold, indicating a clear linewidth narrowing at threshold

☐ Yes ☒ No — Lasing experiments are not involved in this work.

Resolution of the spectrometer used to make spectral measurements

☐ Yes ☒ No — Lasing experiments are not involved in this work.

3. Coherent emission

Measurements of the coherence and/or polarization of the emission

☐ Yes ☒ No — Lasing experiments are not involved in this work.

4. Beam spatial profile

Image and/or measurement of the spatial shape and profile of the emission, showing a well-defined beam above threshold

☐ Yes ☒ No — Lasing experiments are not involved in this work.

5. Operating conditions

Description of the laser and pumping conditions
Continuous-wave, pulsed, temperature of operation

☐ Yes ☒ No — Lasing experiments are not involved in this work.

Threshold values provided as density values (e.g. W cm^{-2} or J cm^{-2}) taking into account the area of the device

☐ Yes ☒ No — Lasing experiments are not involved in this work.

6. Alternative explanations

Reasoning as to why alternative explanations have been ruled out as responsible for the emission characteristics
e.g. amplified spontaneous, directional scattering; modification of fluorescence spectrum by the cavity

☐ Yes ☒ No — Lasing experiments are not involved in this work.

7. Theoretical analysis

Theoretical analysis that ensures that the experimental values measured are realistic and reasonable
e.g. laser threshold, linewidth, cavity gain-loss, efficiency

☐ Yes ☒ No — Lasing experiments are not involved in this work.

8. Statistics

Number of devices fabricated and tested

☐ Yes ☒ No — Lasing experiments are not involved in this work.

Statistical analysis of the device performance and lifetime (time to failure)

☐ Yes ☒ No — Lasing experiments are not involved in this work.
